# Lack of Association Between COL1A1 rs1800012 Polymorphism and Anterior Open Bite Malocclusion in a Turkish Case–Control Cohort

**DOI:** 10.3390/genes16101122

**Published:** 2025-09-23

**Authors:** Tolga Polat, Özlem Özge Yılmaz, Elvan Önem Özbilen, Beste Tacal Aslan

**Affiliations:** 1 Department of Basic Medical Sciences, Faculty of Dentistry, Marmara University, 34854 Istanbul, Türkiye; tolga.polat@marmara.edu.tr (T.P.); ozlem.ozge@marmara.edu.tr (Ö.Ö.Y.); 2 Department of Basic Medical Sciences, Institute of Health Sciences, Marmara University, 34854 Istanbul, Türkiye; 3 Department of Orthodontics, Faculty of Dentistry, Marmara University, 34854 Istanbul, Türkiye; eozbilen@marmara.edu.tr

**Keywords:** open bite, *COL1A1*, genetic polymorphism, malocclusion, genetic

## Abstract

Background/Objectives: Anterior open bite is a multifact orial malocclusion influenced by genetic and environmental factors. Variants in the Collagen type I, alpha 1 (*COL1A1*) gene, particularly rs1800012, have been implicated in bone quality, but their role in craniofacial anomalies remains unclear. Methods: A case–control study was conducted with 60 participants (30 anterior open bite cases; 30 matched controls). DNA was extracted from buccal swabs, and rs1800012 genotyping was performed using TaqMan assays. Genotype and allele distributions were compared with chi-square and Fisher’s exact tests; Hardy–Weinberg equilibrium was assessed in controls. Results: Genotype (GG/GT/TT: 53.3/40.0/6.7% vs. 60.0/33.3/6.7%) and allele (T allele: 26.7% vs. 23.3%) frequencies did not differ significantly between cases and controls. No association was detected under additive, dominant, or recessive models (all *p* > 0.05). Wide confidence intervals indicated limited precision of effect estimates. Conclusions: This study provides no evidence of association between *COL1A1* rs1800012 and anterior open bite in this Turkish cohort. The relatively small sample size, the rarity of the TT genotype, and the multifactorial nature of craniofacial development represent important limitations. Larger, multi-gene, and functionally integrated studies are required to clarify the genetic architecture of open bite malocclusion.

## 1. Introduction

Anterior open bite malocclusion is a dental and skeletal condition characterized by the lack of vertical overlaps between the maxillary and mandibular anterior teeth when the posterior teeth are in occlusion. This condition affects both esthetics and function, leading to issues such as speech impediments, difficulty in mastication, and altered facial morphology [1]. Open bite can be classified as skeletal or dental, depending on whether the underlying cause is related to craniofacial bone structure or dentoalveolar positioning [2]. While parafunctional habits such as thumb sucking, tongue thrusting, and prolonged pacifier use are commonly associated with open bite etiology, genetic predisposition also plays a critical role in its development [3].

The completion of the Human Genome Project (HGP) has significantly advanced our understanding of genetic variations and their implications in various disorders, including craniofacial anomalies. One of the most widely studied genetic variations is the single nucleotide polymorphism (SNP), which represents a single base-pair change in the DNA sequence [4]. SNPs have been identified as important genetic markers in understanding disease susceptibility, gene regulation, and individual responses to environmental factors [5].

In the field of orthodontics and craniofacial genetics, SNPs have been associated with variations in maxillofacial growth, tooth development, and malocclusion patterns. Several genes, including those encoding extracellular matrix proteins, have been implicated in craniofacial development, emphasizing the role of collagen in bone and connective tissue integrity [6,7].

Collagen is the most abundant structural protein in the human body and a key component of connective tissues, including bone, skin, tendons, and ligaments. Type I collagen, encoded by the *COL1A1* and *COL1A2* genes, is essential for maintaining the biomechanical properties of bone and dentoalveolar structures [8,9]. Mutations or polymorphisms in *COL1A1* have been linked to various skeletal disorders, including osteoporosis and osteogenesis imperfecta [10]. These genetic alterations can lead to defects in collagen synthesis, affecting bone mineral density and structural integrity. Recent studies have identified specific *COL1A1* and *COL1A2* mutations that contribute to the severity of osteogenesis imperfecta, particularly in types I and III, highlighting the role of extracellular matrix proteins in skeletal homeostasis [11].

The *COL1A1* gene, located on chromosome 17q21.33, encodes the pro-alpha1 chain of type I collagen, which undergoes post-translational modifications to form mature collagen fibrils. Genetic variations in *COL1A1*, particularly SNPs, can alter collagen synthesis, stability, and function, thereby influencing skeletal development and integrity [12]. Recent studies have shown that specific SNPs within the *COL1A1* gene impact the production ratio of collagen α1(I) and α2(I) chains, potentially reducing bone strength and increasing susceptibility to musculoskeletal disorders and injuries [13].

Among the identified polymorphisms in *COL1A1*, the rs1800012 variant is one of the most extensively studied due to its role in bone metabolism and skeletal strength. This SNP involves a G to T substitution in the first intron of the gene, affecting the binding affinity of the Sp1 transcription factor, which regulates COL1A1 expression [14]. Studies suggest that the T allele may lead to an altered *COL1A1/COL1A2* ratio, resulting in reduced collagen fibril stability and increased bone fragility [15]. Recent research highlights the influence of rs1800012 on bone mineral density and its potential contribution to osteoporosis risk, particularly in postmenopausal women and individuals with genetic predisposition to low bone mass [16]. Additionally, bioinformatics analyses have confirmed that variations at the Sp1 binding site impact *COL1A1* transcription efficiency, further supporting the association between this polymorphism and skeletal fragility [17].

While rs1800012 has been associated with osteoporosis and other bone-related conditions, its potential role in craniofacial growth and malocclusions remains unclear. Given that collagen type I is a major component of the craniofacial skeleton, it is hypothesized that variations in *COL1A1* may contribute to alterations in maxillary and mandibular development, potentially predisposing individuals to open bite malocclusion [18].

Understanding the genetic basis of open bite malocclusion is essential for developing personalized treatment strategies and early intervention approaches. Given the structural role of collagen in bone formation, it is plausible that the *COL1A1* rs1800012 polymorphism could influence craniofacial morphology and occlusal relationships. Recent studies suggest that variations in *COL1A1*, particularly rs1800012, may contribute to altered mandibular growth and facial asymmetry, which are commonly associated with malocclusions, including anterior open bite [19]. Additionally, genetic analyses indicate that individuals carrying specific *COL1A1* polymorphisms exhibit variations in occlusal patterns, supporting the hypothesis that collagen-related genes play a role in craniofacial development and dental arch alignment [20].

The biomechanical properties of collagen type I contribute to the rigidity and resilience of craniofacial structures. If a genetic variation in *COL1A1* results in reduced collagen integrity, it may impact the vertical development of the maxilla and mandible, predisposing individuals to skeletal open bite. Additionally, *COL1A1* polymorphisms may interact with other genes involved in bone remodeling, further complicating the etiology of malocclusion [21].

Previous studies have suggested that *COL1A1* variants play a role in craniofacial morphology, but the specific link between rs1800012 and open bite malocclusion remains underexplored [5]. This study aims to bridge this gap by investigating whether COL1A1 rs1800012 polymorphism is associated with open bite malocclusion and whether its presence correlates with variations in occlusal patterns. Recent findings suggest that rs1800012 may influence mandibular growth and facial morphology, potentially contributing to skeletal discrepancies observed in malocclusion cases [19]. Furthermore, genetic analyses indicate that individuals with this polymorphism exhibit distinct occlusal characteristics, reinforcing the hypothesis that collagen-related gene variants impact craniofacial structure and dental alignment [20].

By integrating genetic analysis into orthodontic research, we can enhance our understanding of malocclusion etiology and improve treatment modalities through a more targeted and individualized approach.

## 2. Material and Methods

### 2.1. Power Analysis Data

The sample size for our study was determined by G*Power 3.1.9.4 software, based on the alpha criterion of the hypothesis and its effect size. We followed the study by Küchler and collaborators, according to the OR value indicating that the GG genotype of the MMP-9 gene reduces open bite risk (OR = 0.18, 95% CI: 0.01–1.79) against the control group (α = 0.05, 1-β = 0.85, that is, error 0.05 and test power 95%) with 30 samples per group [22]. Thus, the same strategy was followed in our study when calculating the sample size for *COL1A1* rs1800012 polymorphism. We acknowledge this as a limitation; sample size estimation was based on prior SNP studies, but power for rs1800012 was limited due to TT rarity.

### 2.2. Study Design and Participants

This case–control genetic association study was conducted at the Faculty of Dentistry, Marmara University (Istanbul, Türkiye). The protocol adhered to the Declaration of Helsinki and received approval from the Marmara University Faculty of Medicine Clinical Research Ethics Committee (Protocol code: 09.2022.652; approval date: 13 June 2022). Written informed consent was obtained from adult participants and from parents/guardians for minors prior to any procedure.

Consecutive patients aged 15–35 years who presented to the Orthodontics Department were screened. Inclusion for cases required a clinical diagnosis of anterior open bite confirmed on lateral cephalograms. Controls were age- and sex-matched individuals without open bite or prior orthodontic treatment that could affect vertical measurements. Exclusion criteria were syndromic conditions, craniofacial trauma, systemic bone diseases, prior orthognathic surgery, and poor-quality radiographs. All participants self-reported Turkish ancestry for at least two generations to reduce (but not eliminate) the risk of population stratification.

Anterior open bite was defined clinically as zero or negative vertical overlap of maxillary and mandibular incisors in centric occlusion. To distinguish dental from skeletal open bite, standardized lateral cephalograms (natural head posture, teeth in intercuspation) were traced in NemoStudioNX-Pro (v10.4.2; Software Nemotec, Madrid, Spain). In addition to overbite (mm), vertical and sagittal skeletal parameters were measured: SN–MP (°), FMA (°), SN–GoGn (°), SNA (°), SNB (°), ANB (°), and PFH/AFH ratio. Skeletal open bite was defined by negative/zero overbite accompanied by a hyperdivergent vertical pattern (elevated SN–MP and/or SN–GoGn) consistent with established orthodontic standards, while dental open bite had normal vertical skeletal indices with dentoalveolar features.

For within-case exploratory analyses, severity was categorized using overbite (mm) cut-points aligned with clinical practice and the prior orthodontic literature: mild (0 to −0.9 mm), moderate (−1.0 to −4.9 mm), and severe (≤−5.0 mm). Categories were corroborated by the cephalometric profile.

To minimize sparse cells, severity analyses were treated as exploratory and are complemented by ordinal models. (Table S1).

### 2.3. Sample Collection and Genotyping

The isolation of DNA from buccal epithelial cells was conducted in accordance with the PureLink protocol (Invitrogen, Van Allen Way, Carlsbad, CA, USA). The quantity of isolated DNA was subsequently measured using the Qubit 4 fluorometer, in accordance with the manufacturer’s protocol.

Genotyping was performed on a StepOnePlus Real-Time PCR System (Thermo Fisher Scientific, Waltham, CA, USA) using TaqMan^®^ SNP Genotyping Assays (cat. no. 4351379). Allelic discrimination followed the manufacturer’s guidelines; cluster plots were visually inspected. To avoid sequence-level transcription errors in the main text, probe/primer IDs are provided in Table S2. Ten percent of samples were genotyped in duplicate (blinded) and all plates included no-template controls. Allele calling was exported as GG/GT/TT for rs1800012 (G>T in intron 1; Sp1 site).

### 2.4. Statistical Analysis

Statistical analyses were performed using SPSS v25.0 (IBM, Armonk, NY, USA). Genotype and allele distributions between cases and controls were compared using Pearson’s chi-square or Fisher’s exact tests, as appropriate. Hardy–Weinberg equilibrium (HWE) was assessed in the control group by exact test.

Exploratory subgroup analyses were conducted by stratifying cases into mild, moderate, and severe open bite categories based on cephalometric overbite values; genotype distributions across subgroups were compared descriptively with chi-square tests.

Although logistic regression is generally recommended for genetic association studies, the very small number of TT homozygotes (*n* = 2 per group) resulted in sparse cells and unstable estimates with excessively wide confidence intervals. To avoid misleading effect sizes, only chi-square/Fisher’s exact results are reported here. Effect sizes are expressed as odds ratios (ORs) with 95% confidence intervals where estimable. A two-sided *p* < 0.05 was considered statistically significant.

## 3. Results

Genotype distributions for *COL1A1* rs1800012 were similar between the open bite and control groups, with no significant differences observed (*p* > 0.05). The allele frequency analysis also showed no significant association between the rs1800012 polymorphism and open bite severity.

The genotype distribution of the COL1A1 rs1800012 polymorphism in the open bite group was GG: 53.3%, GT: 40.0%, and TT: 6.7%, whereas in the healthy control group it was GG: 60.0%, GT: 33.3%, and TT: 6.7%. Chi-square analysis revealed no statistically significant differences in genotype distribution between the two groups (*p* = 0.8609). Similarly, allele frequency analysis showed that the G allele was observed at a frequency of 73.3% in the open bite group and 76.7% in the control group, while the T allele was present at 26.7% and 23.3%, respectively. These differences were not statistically significant (*p* = 0.6733).

Hardy–Weinberg equilibrium (HWE) analysis demonstrated that both groups were in genetic equilibrium, with *p*-values of 0.992 for the patient group and 0.932 for the control group. This indicates that the observed genotype frequencies are consistent with expected population genetic equilibrium, supporting the reliability of the sample without evidence of genotyping error or population stratification.

Taken together, these findings suggest that the rs1800012 polymorphism does not exert a significant influence on genetic susceptibility to open bite malocclusion. The lack of statistically significant differences indicates that this SNP may not play a decisive role in determining vertical occlusal relationships in the studied population.

In the genotypic dominance analysis of rs1800012 polymorphism, no statistically significant differences were observed in the comparisons between the TT genotype and other genotypes (TT vs. GG; TT vs. GG + GT; TT vs. GT). The obtained odds ratios (ORs) were 1.12, 1.00, and 0.83, respectively; however, all confidence intervals were wide and included the null value of 1. Furthermore, the *p*-values were 1.000 across all comparisons, indicating no significant association between the rs1800012 polymorphism and open bite malocclusion in terms of genotypic dominance (Table 1). These findings also suggest that the limited sample size may have reduced statistical power, and that this polymorphism does not contribute meaningfully to open bite susceptibility in the studied population.

**Table 1:** *COL1A1* rs1800012 genotype, allele, and model distributions in anterior open bite and controls.

Model/Measure	Cases (*n* = 30)	Controls (*n* = 30)	HWE *p* (Controls)	OR (95% CI) †	*p*-Value
Genotype counts (%)	GG: 16 (53.3%) GT: 12 (40.0%) TT: 2 (6.7%)	GG: 18 (60.0%) GT: 10 (33.3%) TT: 2 (6.7%)	0.932	–	χ^2^ = 0.861
Allele counts (%)	G: 44 (73.3%) T: 16 (26.7%)	G: 46 (76.7%) T: 14 (23.3%)	–	OR = 1.19 (0.52–2.73)	0.673
Additive model (per T allele)	–	–	–	OR = 1.19 (0.52–2.73)	0.673
Dominant model (GT + TT vs. GG)	14 vs. 16	12 vs. 18	–	OR = 1.31 (0.47–3.65)	0.59
Recessive model (TT vs. GG + GT)	2 vs. 28	2 vs. 28	–	OR = 1.00 (0.13–7.60)	1.00

† Odds ratios (ORs) and 95% confidence intervals (CIs) estimated where counts allowed. HWE: Hardy–Weinberg equilibrium in controls. Statistical significance was evaluated at *p* < 0.05. Comparison between the healthy and patient groups was performed using the χ^2^ test. Significance indicates *p* < 0.05.

The open bite group was further categorized based on severity: mild (0 to −0.9 mm), moderate (−1 to −4.9 mm), and severe (−5 mm or greater). The distribution of COL1A1 rs1800012 genotypes within these subgroups did not show any significant correlation with severity levels (*p* > 0.05). There were no statistically significant variations in genotype distribution, suggesting that this SNP does not contribute to progressive worsening of open bite severity. This finding implies that the COL1A1 rs1800012 polymorphism may not be a major genetic determinant of open bite severity. Future studies with larger sample sizes and functional analyses are needed to further investigate the potential role of this polymorphism in craniofacial development and occlusal anomalies.

The analysis of COL1A1 rs1800012 allelic distribution in the patient group reveals no statistically significant association between allele frequency and open bite severity (*p* = 0.1513). The G allele was more prevalent across all severity groups, comprising 73.3% of the total allele count, whereas the T allele accounted for 26.7%. There was no statistically significant difference in the G allele frequency observed in individuals with moderate (−1 to −4.9 mm) and severe (−5 mm and above) open bite categories (Table 2).

**Table 2:** Distribution of *COL1A1* rs1800012 genotypes and allele frequencies according to anterior open bite severity in patients.

Overbite Severity (mm)	Genotype	N (Individuals)	Genotype Frequency (%)	Within Group (%)	*p*-Value (χ^2^ Test)
0–0.9 mm	GG	5	31.2%	38.5%	0.3239
	GT	6	50.0%	46.1%	
	TT	2	100.0%	15.4%	
−1 to −4.9 mm	GG	8	50.0%	72.7%	
	GT	3	25.0%	27.3%	
	TT	0	0.0%	0.0%	
−5 mm and above	GG	3	18.8%	50.0%	
	GT	3	25.0%	50.0%	
	TT	0	0.0%	0.0%	
Total	GG	16	53.3%	100.0%	
	GT	12	40.0%	100.0%	
	TT	2	6.7%	100.0%	
Allelic Frequency					
0–0.9 mm	G	16	36.4%	61.5%	0.1513
	T	10	62.4%	38.5%	
−1 to −4.9 mm	G	19	43.2%	86.4%	
	T	3	18.8%	13.6%	
−5 mm and above	G	9	42.4%	75.0%	
	T	3	18.8%	25.0%	
Total	G	44	73.3%	100.0%	
	T	16	26.7%	100.0%	

Note: Chi-square (χ^2^) test was used to compare genotype distributions among different overbite severity groups. A *p*-value < 0.05 was considered statistically significant. The χ^2^ test values and *p*-values for individual comparisons are provided in the table.

## 4. Discussion

In this case–control cohort, no evidence of association was found between *COL1A1* rs1800012 and anterior open bite malocclusion. Genotype and allele frequencies did not differ significantly between cases and controls, and no association was observed across severity subgroups. These findings suggest that rs1800012 alone does not exert a strong effect on the etiology of anterior open bite.

Previous studies on collagen-related polymorphisms and craniofacial anomalies have yielded inconsistent results. For example, *COL1A1* rs2249492 has been linked to Class III malocclusions, and *FGFR2* rs2981582 has been associated with both Class II and Class III malocclusions [18]. In contrast, other investigations have reported no significant associations, similar to our findings for rs1800012. Moreover, variants in genes such as *MMP-1* rs1799750 have been implicated in extracellular matrix remodeling and open bite risk [23,24,25], while *ACTN3* rs1815739 has been associated with differences in muscle function and a higher prevalence of the XX genotype among individuals with open bite [26]. These discrepancies highlight the multifactorial and polygenic basis of malocclusion, in which single-SNP effects are insufficient to explain phenotypic variability.

*COL1A1* encodes the α1 chain of type I collagen, essential for bone strength, tendon structure, and craniofacial integrity. Mutations in this gene cause osteogenesis imperfecta and Ehlers–Danlos syndrome, both characterized by skeletal anomalies [1]. Jabbour et al. (2018) also reported that collagen chain mutations may influence malocclusion severity in OI patients [27]. While such findings underscore the biological relevance of *COL1A1*, common variants like rs1800012 likely exert subtle or indirect effects, if any, on craniofacial morphology. It remains plausible that *COL1A1* interacts with other matrix-related genes (e.g., *MMP*s, *COL1A2*) and muscle-related loci (e.g., ACTN2, ACTN3) [28], but our study did not detect a direct contribution of rs1800012 to open bite.

Our results support the view that anterior open bite arises from a combination of genetic predispositions and environmental influences. “The absence of association between *COL1A1* rs1800012 and anterior open bite in our cohort should be interpreted with caution, as several methodological and biological explanations may account for this negative finding”.

First, the limited sample size (*n* = 60) and, more importantly, the very low frequency of the TT genotype (2 individuals per group) substantially reduced the statistical power of our analyses. Under these conditions, only large effect sizes could be detected, whereas subtle or moderate genetic influences would remain undetected. This constraint is consistent with previous studies reporting that single-SNP effects in complex craniofacial traits generally require several hundred participants to achieve adequate statistical power.

Second, anterior open bite represents a heterogeneous phenotype. While we distinguished between dental and skeletal open bite using standardized cephalometric parameters, genetic contributions may differ across these subtypes. Skeletal open bite is more likely to involve craniofacial developmental pathways, whereas dental open bite may be strongly influenced by dentoalveolar compensations and oral habits. Grouping them together may dilute potential associations, particularly in a small sample.

Third, rs1800012 may exert its effects not in isolation, but through interactions with other genetic loci. Candidate genes such as *COL1A2*, matrix metalloproteinases (*MMP-1*, *MMP-9*), and muscle-related genes (*ACTN2*, *ACTN3*) have been implicated in craniofacial morphology and malocclusion risk. Epistatic interactions among collagen-related genes could modulate bone quality and maxillofacial growth, meaning that evaluating only rs1800012 may not capture the polygenic nature of anterior open bite.

Fourth, environmental influences may overshadow subtle genetic effects in this condition. Parafunctional habits such as thumb sucking, tongue thrusting, and prolonged pacifier use, as well as airway obstruction and altered tongue posture, are well-documented contributors to open bite development. It is plausible that in our cohort these environmental determinants exerted stronger effects than rs1800012 variation.

Fifth, allele frequencies and linkage disequilibrium patterns of rs1800012 can vary across populations. Our study was restricted to a single Turkish cohort, which limits generalizability and raises the possibility that rs1800012 contributes differently in other ethnic groups. Larger, multi-center studies are necessary to clarify potential population-specific effects.

Taken together, these factors help explain why we found no significant association between COL1A1 rs1800012 and anterior open bite. Importantly, our findings should not be interpreted as definitive evidence of absence of effect, but rather as no evidence of association within the constraints of this study. This highlights the need for larger sample sizes, multi-gene panels, and integrative approaches combining genetic, environmental, and functional analyses to fully elucidate the complex etiology of anterior open bite. Additionally, because multiple genetic models (additive, dominant, recessive) were tested, we applied Bonferroni correction to minimize type I error risk. After adjustment, no association remained significant, reinforcing that our findings reflect a genuine lack of association rather than a false positive result.

Previous reports emphasize the role of oral habits, tongue posture, and airway patterns in shaping vertical skeletal growth [21,29]. Additionally, differences in cephalometric features between open bite and deep bite cases provide further evidence of the heterogeneity of this malocclusion [25,30]. The significant impact of open bite on oral function, esthetics, and psychosocial well-being has also been documented [29], reinforcing the need for early diagnosis and comprehensive treatment strategies.

Several limitations should be acknowledged. First, the sample size (*n* = 60) limited the precision of effect estimates and prevented reliable regression modeling due to sparse TT counts. As such, the study was able to rule out large effects but not modest ones. Second, the study population was restricted to a single Turkish cohort, limiting generalizability and raising the possibility of population-specific effects. Although we required self-reported Turkish ancestry for at least two generations to reduce heterogeneity, no genetic methods such as principal component analysis were performed to formally control for population stratification. Therefore, subtle background population structure cannot be completely excluded as a potential confounder. Third, only rs1800012 was analyzed, whereas other loci in COL1A1, COL1A2, FGFR2, and genes related to bone metabolism and muscle function may contribute more substantially. Finally, functional assays of collagen metabolism were not performed, so biological inference remains indirect.

Future studies should therefore recruit larger, multi-center cohorts with greater ethnic diversity, evaluate broader SNP panels or genome-wide markers, and incorporate longitudinal designs to explore genetic influences on treatment response. Functional analyses of COL1A1 and related pathways will also be important to clarify biological mechanisms. Integration of cephalometric assessments with gene–environment interactions (oral habits, airway, muscle function) will be essential to advance the understanding of anterior open bite etiology.

## 5. Conclusions

In conclusion, this pilot case–control study found no evidence of association between *COL1A1* rs1800012 polymorphism and anterior open bite malocclusion in a Turkish cohort. Genotype and allele distributions were similar between cases and controls, and no statistically significant differences were detected across severity subgroups. These findings suggest that rs1800012 alone does not play a decisive role in the susceptibility to anterior open bite. Nevertheless, the results must be interpreted with caution due to several important limitations, including the relatively small sample size, the very low frequency of the TT genotype, the restriction to a single population, and the analysis of only one SNP. Under these conditions, strong genetic effects appear unlikely, but subtle or context-dependent contributions cannot be excluded.

Importantly, anterior open bite represents a multifactorial malocclusion influenced by both genetic predispositions and environmental determinants. A single-SNP approach, while valuable for generating preliminary insights, is insufficient to capture the polygenic and heterogeneous nature of this condition. Thus, our results should be considered as preliminary evidence that highlights the need for broader genetic research rather than definitive conclusions.

Future studies should therefore recruit larger and ethnically diverse cohorts, apply standardized cephalometric criteria to reduce phenotypic heterogeneity, and evaluate extended panels of candidate genes alongside gene–gene and gene–environment interactions. Incorporating functional analyses such as collagen expression assays, biomechanical testing, and transcriptomic profiling will also be essential to establish potential biological mechanisms. Integrative approaches that combine genetic data with clinical and environmental parameters hold promise for unraveling the complex etiology of anterior open bite. A more comprehensive genetic understanding of this malocclusion will ultimately support earlier and more accurate diagnosis, enable personalized orthodontic treatment planning, and improve long-term therapeutic outcomes.

## Data Availability

The data supporting the findings of this study are available from the corresponding author upon reasonable requests. Owing to institutional policies and ethical restrictions, the dataset is not publicly available.

## Supplementary Materials

The following supporting information can be downloaded at: https://www.mdpi.com/article/10.3390/genes16101122/s1, Table S1. Classification of open bite patients based on overbite severity (mm); Table S2. Sequences of TaqMan probe used for genotyping of *COL1A1* rs1800012 polymorphism.

## Abbreviations

The following abbreviations are used in this manuscript:

ACTN2	Alpha-Actinin-2
ACTN3	Alpha-Actinin-3
CI	Confidence Interval
COL1A1	Collagen Type I Alpha 1
COL1A2	Collagen Type I Alpha 2
DNA	Deoxyribonucleic Acid
EDS	Ehlers–Danlos Syndrome
FGFR2	Fibroblast Growth Factor Receptor 2
HGP	Human Genome Project
MMP-1	Matrix Metalloproteinase-1
MMP-9	Matrix Metalloproteinase-9
OI	Osteogenesis Imperfecta
OR	Odds Ratio
PCR	Polymerase Chain Reaction
qPCR (Real-Time PCR)	Quantitative Polymerase Chain Reaction
SNP	Single Nucleotide Polymorphism
Sp1	Specificity Protein 1 (transcription factor)
SPSS	Statistical Package for the Social Sciences
