# Supplementary material for: Lack of Association Between COL1A1 rs1800012 Polymorphism and Anterior Open Bite Malocclusion in a Turkish Case–Control Cohort
Source: Genes (Basel). 2025 Sep 23;16(10):1122. doi: 10.3390/genes16101122 (PMC12562384; doi:10.3390/genes16101122)
Supplement: Supplementary file 1 [file genes-16-01122-s001.zip › genes-3882921-supplementary.pdf]

Lack of Association Between COL1A1 rs1800012 Polymorphism and Anterior Open Bite Malocclusion in a Turkish Case–Control Cohort

Tolga Polat [1,2], Özlem Özge Yılmaz [1,2], Elvan Önem Özbilen [3] and Beste Tacal Aslan [1,*]

1 Department of Basic Medical Sciences, Faculty of Dentistry, Marmara University, 34854 Istanbul, Türkiye
2 Department of Basic Medical Sciences, Institute of Health Sciences, Marmara University, 34854 Istanbul, Türkiye
3 Department of Orthodontics, Faculty of Dentistry, Marmara University, 34854 Istanbul, Türkiye
* Correspondence: btacal@gmail.com

Table of contents

Table S1. Classification of open bite patients based on overbite severity (mm). 3

Table S2. Sequences of TaqMan probe used for genotyping of *COL1A1* rs1800012 polymorphism... 3

Table S1. Classification of open bite patients based on overbite severity (mm).

	Open bite Patient Group		
	**Group 1**	**Group 2**	**Group 3**
**Overbite**	0 and -0.9 mm	-1 and -4.9mm	-5 and above
**n**	13	11	6

Table S2. Sequences of TaqMan probe used for genotyping of *COL1A1* rs1800012 polymorphism.

		DNA sequence (5'→3')
**VIC/FAM**	***COL1A1* rs1800012**	GGGAGGTCCAGCCCTCATCCCGCCC**[A/C]**CATTCCCTGGGCAGGTGGGGTGGCG
